# L1CAM expression in endometrial carcinomas is regulated by usage of two different promoter regions

**DOI:** 10.1186/1471-2199-11-64

**Published:** 2010-08-27

**Authors:** Marco Pfeifer, Uwe Schirmer, Claudia Geismann, Heiner Schäfer, Susanne Sebens, Peter Altevogt

**Affiliations:** 1 German Cancer Research Center, Department of Translational Immunology, D015, D-69120 Heidelberg, Germany; 2 Clinic for Internal Medicine I, Laboratory of Molecular Gastroenterology and Hepatology, UKSH-Campus, University of Kiel, D- 21405 Kiel, Germany

## Abstract

**Background:**

The L1 cell adhesion molecule (L1CAM) was originally identified as a neural adhesion molecule involved in axon guidance. In many human epithelial carcinomas L1CAM is overexpressed and thereby augments cell motility, invasion and metastasis formation. L1CAM positive carcinomas are associated with bad prognosis. Recent data point out that L1CAM is regulated in a fashion similar to epithelial-mesenchymal transition (EMT). Previous studies have implied the transcription factors Slug and/or β-catenin in *L1CAM *transcriptional regulation. However, the regulation of human L1CAM expression at the transcriptional level is not well understood.

**Results:**

To better understand the molecular basis of *L1CAM *transcriptional regulation, we carried out a detailed characterization of the human *L1CAM *promoter. We identified two transcription start sites, the first in front of a non-translated exon 0 (promoter 1) and the other next to the first protein-coding exon 1 (promoter 2). Both sites could be verified in endometrial carcinoma (EC) cell lines and appear to be used in a cell-type specific manner. The two identified promoter regions showed activity in luciferase reporter assays. Chromatin-IP analyses confirmed the *in silico *predicted E-boxes, binding sites for transcription factors Snail and Slug, as well as Lef-1 sites, which are related to β-catenin-mediated transcriptional regulation, in both promoters. Overexpression of β-catenin exclusively augmented activity of promoter 1 whereas Slug enhanced promoter 1 and 2 activity suggesting that both promoters can be active. Overexpression of β-catenin or Slug could upregulate L1CAM expression in a cell-type specific manner.

**Conclusions:**

Our results, for the first time, provide evidence that the L1CAM gene has two functionally active promoter sites that are used in a cell-type specific manner. Slug and β-catenin are involved *L1CAM *transcriptional regulation. Nevertheless, Slug rather than β-catenin levels are correlated with L1CAM expression in EC cell lines. Our findings suggest that the *L1CAM *transcriptional regulation is more complex than anticipated and this study provides the basis for a better understanding of L1CAM regulation in non-neuronal/tumor cells.

## Background

The integrity and plasticity of normal epithelial cell layers is tightly controlled by cell-cell contacts mediated by cell surface receptors that are collectively referred to as cell adhesion molecules. The breakdown of epithelial cell homeostasis during aggressive cancer progression is correlated with loss of epithelial characteristics and frequently leads to a disregulated expression of cell adhesion receptors. A well-studied example is the loss of E-Cadherin expression especially in adherens junctions during epithelial-mesenchymal transition that is thought to precede the onset of tumour metastasis [1,2].

The neural cell adhesion molecule L1CAM plays a fundamental role in the development of the nervous system [3,4]. Whereas in normal epithelium the L1CAM expression is very low and hardly detectable, this changes after neoplastic transformation. Indeed, overexpression of L1CAM has been reported in carcinomas such as ovarian and endometrial, colon, pancreas, kidney, cholangiocarcinoma, gastric cancer but also melanoma [5-9]. Wherever investigated, the expression of L1CAM was associated with bad prognosis suggesting that, directly or indirectly, L1CAM drives tumour progression. The mechanisms by which L1CAM mediates these effects are not clearly established. But work from experimental systems showed that L1CAM augments tumour growth in NOD/SCID mice, enhances cell motility on extracellular matrix proteins and increases matrigel invasion [10-13]. Other studies reported L1CAM-dependent gene expression signatures, metastasis formation [13-15] and an augmented resistance to apoptotic stimuli [16,17]. This raises the important question how L1CAM expression is regulated in human tumours.

The *L1CAM *gene is located at chromosome Xq28 spanning about 25 kb with 28 coding exons [18,19]. Most insights into the *L1CAM *gene organisation and regulatory elements were obtained in the field of neurobiology. Initial work on the organization of the 5'-end of the gene has placed a transcription initiation site in front of exon 1 that encodes the ATG in adult mouse brain and N2A neuroblastoma cells [20]. A fragment encompassing this region displayed promoter activity but a second promoter was suggested > 5kb upstream of the latter site [20]. Subsequent work has confirmed the presence of a promoter element more than 10 kb upstream, in front of the non-translated exon 0 [21]. Importantly, the existence of a second transcription start site (TSS) in front of exon 1 was put into question. The organisation of the *L1CAM *gene was found to be similar between human and mouse [21].

In immunohistochemical sections, L1CAM expression is often seen at the invasive front where the tumour invades into the surrounding stroma [12,22-24]. Cells at the invasive front are often enriched for nuclear β-catenin localisation in contrast to the more central tumour areas, e.g. in colon tumours [25]. Indeed, *L1CAM *was identified as a target gene of the Wnt/β-catenin signalling pathway [12] and nuclear β-catenin was shown to co-localise with L1CAM [23]. This work has suggested that the β-catenin/TCF-LEF transcriptional complex may be an important direct regulator of L1CAM expression. Recent studies have shown that an alternative pathway of *L1CAM *transcriptional regulation may exist. The treatment of endometrial carcinoma cells with the EMT-inducer TGF-β1 augmented L1CAM expression and downregulation of E-Cadherin and this process was blocked by knockdown of Slug [24,26]. In pancreatic carcinoma cells TGF-β1 treatment also upregulated L1CAM and that was prevented by knockdown of Slug [26]. Thus, β-catenin/TCF-LEF and Slug are implicated in the transcriptional regulation of L1CAM but it is unknown how this is coordinated.

The promoter organisation of the human *L1CAM *gene is not fully understood. We therefore intended to clarify the transcriptional regulation of *L1CAM *as well as whether there are alternative promoter regions. In particular, we analysed binding sites for the transcription factors β-catenin/TCF-LEF and Slug in the human promoter. We provide evidence for two TSS and two active elements in the human *L1CAM *promoter. Promoter 1 and promoter 2 are differentially used in human endometrial cell lines. Whereas promoter 1 is most responsive to β-catenin overexpression and to a lesser extent by Slug, promoter 2 is activated only by Slug. Our results provide the basis for a better understanding of *L1CAM *regulation in tumours.

## Methods

### Cell lines, cell culture and transfections

ECC-1 and Hec-1A cells were maintained in DMEM/F12 medium (PAA Laboratories, Pasching, Austria) supplemented with 10% fetal calf serum, Hec-1B and SPAC-1L cells in RPMI-1640 (PAA Laboratories, Pasching, Austria) with 10% fetal calf serum at 37°C, 5% CO2 and 100% humidity. All other endometrial carcinoma cell lines were described before [24,27]. Transient transfection of ECC-1 and Hec-1A cells was done using jetPEI (Polyplus, Illkirch, F). 1x10^5 ^cells were seeded 24 h before transfection in 6-well plates. For luciferase assays cells were transfected with 2 μg of the corresponding firefly-luciferase-construct (in pGl3-basic vector), 20 ng of renilla luciferase and if indicated 1 μg Snail-HA, Slug-HA or β-catenin construct, respectively. (Snail-HA and Slug-HA constructs were kindley provided by Dr. Herreros, Barcelona, Spain). The β-catenin (S33Y) plasmid was a gift of Dr. Avri BenZeev, Weizmann Institute, Rehovot, Israel. The transfections were done as indicated in the manufacturer's protocol. For ChIP assays, cells were seeded in 175 cm^2 ^dishes, transfected either with pcDNA3 control, β-catenin (S33Y) or Slug-HA, respectively. Cells were harvested 72 h after transfection. For induction of EMT cells were cultivated in medium in the presence of TGF-β1(10 ng/ml) for 5 days prior to luciferase transfection.

### Chemicals and antibodies

Antibodies to the ectodomain of L1CAM (monoclonal antibody (mAb) L1-11A, a subclone of UJ127.11), were described before [10,15]. ChIP-grade antibodies against HA, β-catenin and Histone-H3 were obtained from Cell Signaling Technology (Danvers, MA, USA). Antibodies for detection in Western blot against β-catenin were from Sigma-Aldrich (Schnelldorf, Germany) and against GAP-DH from SantaCruz Biotechnology (Heidelberg, Germany).

### Construction of Reporter Plasmids

Promoter-1 constructs: Primers for PCR from a bacterial artificial chromosome (Xq28: *RZPDB737A112189D*) clone were as follows: P1 fw 5'-tagtatACGCGTcaaaggcaggttcaacatca-3'; P2 fw 5'-tagtatACGCGTggcttctgcgtcttctgca-3'; P3 fw 5'-tagtatACGCGTtgggctttggttttctcatc-3'; Promoter-1 rev 5'-tagtatCTCGAGctgcggcagcagcggct-3'. Forward primers contain a MluI site (capital letters) and reverse primer contains an XhoI site (capital letters). PCR fragments were digested and directly cloned into pGl3-basic vector by MluI and XhoI site. Promoter-2 constructs: Primers for PCR from a bacterial artificial chromosome (Xq28: *RZPDB737A112189D*) clone were as follows: L1-A fw 5'-GGTACCttgggaccggacttactcag-3'; L1-B fw 5'-GGTACCgatatgagcctgtggggaga-3'; L1-C fw 5'-GGTACCaactgctgacctcatgatcc-3'; L1-D fw 5'-GGTACCgcagatccacaaccacacac-3'; L1-D* fw 5'-GGTACCgcacatgcagacacatacgg-3'; L1-E fw 5'-GGTACCcgggcttacccagatgttag-3'; L1-F fw 5'-GGTACCttctcccctctcccagtg-3'; Promoter-2 rev 5'-AAGCTTaggagaggccacacgtacc-3'. Forward primers contain a KpnI site (capital letters), reverse primer contains a HindIII site (capital letters). PCR fragments were subcloned in TOPOblunt vector (Invitrogen, Karlsruhe, Germany), digested by HindIII and KpnI and subsequently cloned into pGl3-basic vector. All constructs were sequenced for control purposes.

### Luciferase assay

Cells were lysed 48 h after transfection, and firefly and renilla luciferase (pRL-TK) levels were determined by enzyme assay kit from Promega (Dual luciferase assay kit). Luciferase activity was normalized to renilla activity. Co-transfection with renilla luciferase served as a transfection efficiency control. Activities were calculated as a quotient of firefly by renilla counts and were set in relation to the positive control pGl3-SV40-Luc (100%).

### Quantitative RT- PCR

10 ng of total cDNA were analysed in triplicates. L1CAM, Snail, Slug, and β-catenin specific primers for qPCR were designed with the DNA Star Program and were produced by MWG Eurofines (Ebersberg, Germany). The PCR reaction was performed with the SYBRgreen mastermix (Applied Biosystems, Darmstadt, Germany) in an ABI 7300 analyser. Primers used for determining mRNA expression levels were as follows: L1-CAM fw 5'-ACGAGGGATGGTGTCCACTTCAAA-3'; L1-CAM rev 5'-TTATTGCTGGCAAAGCAGCGGTAG-3'; SNAIL fw 5'-CTGCTCCACAAGCACCAAGAGTC-3'; SNAIL rev 5'-CCAGCTGCCCTCCCTCCAC-3'; SLUG fw 5'-ATATTCGGACCCACACATTACCT-3'; SLUG rev 5'-GCAAATGCTCTGTTGCAGTGA-3'; β-catenin fw 5'-TGCAGTTCGCCTTCACTATGGACT-3'; β-catenin rev 5'-GATTTGCGGGACAAAGGGCAAGAT-3'; and for normalisation β-actin fw 5'-ACAAGATGAGATTGGCATGGC-3'; β-actin rev 5'-GCCACATTGTGAACTTTGGGG-3'. To analyse changes in gene expression in a given sample relative to a reference sample, the comparative Ct method was used as the relative quantification method. For correlation analysis the Δct values were calculated according to Δct = ct(gene of interest)-ct(β-actin). For determination of relative mRNA levels the 2^-ΔΔct ^was calculated where ΔΔct = Δct(b-actin)-Δct(gene).

### Chromatin Immunoprecipitation (ChIP)

For ChIP assays a total number of 3 × 10^7 ^ECC-1 cells were analysed, either transfected with control pcDNA3, Snail-HA or Slug-HA vectors. The ChIP experiments were essentially performed as indicated in the manufacturer's protocol (Cell Signaling, Beverly; USA). In brief, cells were fixed by 1% formaldehyde in PBS. Nuclei were isolated according to the protocol and the chromatin was digested by micrococcus nuclease treatment for 20 min at 37°C, resulting in DNA-fragments of 150-900 bps in length. Subsequent lysis of the nuclei was achieved by sonification. The solution was cleared by centrifugation and the DNA fragments were in the soluble phase. Immunoprecipitation reactions were performed over night at 4°C using antibodies against HA and β-catenin and a rabbit IgG as negative control, and a anti-Histone H3 antibody as a positive control. This was followed by incubation of the immunoprecipitation for another 2 h at 4°C with the protein G magnetic beads. The beads were pelleted by a magnet, several times washed and finally eluted and thereby reverse crosslinked for 30 min at 65°C. The chromatin was finally purified by micro spin columns. 2 μl of the appropriate DNA were analysed by qRT-PCR. All samples were examined in triplicates. The non-immunoprecipitated, so-called input control served as a positive control, another positive control for the efficiency of the procedure is the immunoprecipitation with H3-antibody. As a negative control rabbit IgG was used. E box specific primers for qPCR were designed with the DNA Star Program and were produced by MWG Eurofines (Ebersberg, Germany). Primers used for determining immunoprecipitated chromatin-fragments were as follows: E1 fw 5'-CCCAGAGAAGACACACCACA-3'; E1 rev 5'-CAACTTGTTGCCCCATCA-3'; E2 fw 5'-GCCCAGCCCTAATTTTGTATT-3'; E2 rev 5'-AAAATAAAAGGCCAGGCACA-3'; E3 fw 5'-CCCCCTCTTTCAGACCCTTA-3'; E3 rev 5'-GGGACATTTTCCGTGACAGT-3'; E4 fw 5'-TGGGATTTTCTGGGTGCTT-3'; E4 rev 5'-ACGCAACAGAGCATCAAGG-3'; E5 fw 5'-GTCCACTCAGTGCATGGTCA-3'; E5 rev 5'-GATTCCTGCTGCGGGTAG-3'; E6 fw 5'-AACACGCTGAGGACGAAG-3'; E6 rev 5'-GAAGGGGATGCTCCCTTAG-3'; E7 fw 5'-GTTTCCTCTGCTGTCACCTG-3'; E7 rev 5'-CCATAGTGCCAGCTTCAGTT-3'; E8 fw 5'-GCTGTGCTCAGGAAATGTGA-3'; E8 rev 5'-CCCAACTTCTGCAAATCCTT-3'; E9 fw 5'-GTAACACTCACCGCGAAGGT-3'; E9 rev 5'-TTGAACTCCTGGGCTCAAGT-3'; Lef-1 (1) fw 5'-TACCATCCTTTCGGTGTTCCC-3'; Lef-1 (1) rev 5'-GACAGAGCTTGGAGGCTGAA-3'; Lef-1 (2) fw 5'-AGCTCCTTCCTTCTCGATCCTGT-3'; Lef-1 (2) rev 5'-TTTCCTCCAGCTCTCTGGTGTCTT-3'; Lef-1 (3) fw 5'-TTCTCCTCCTAAAGGCTGGGCAAA-3'; Lef-1 (3) rev 5'-TCGACTCAGTCTCACAAGACTCCC-3'; Lef-1 (4) fw 5'-ACTGAACAATCCACTCCTAAGCGG-3'; Lef-1 (4) rev 5'-TCACTCACTGAAGGACACTTGGGT-3'.

### RNA Ligase Mediated Rapid Amplification of cDNA Ends (RLM-RACE)

Total RNA was isolated using the Qiagen RNeasy mini kit (Qiagen, Hilden, Germany). First Choice RLM-RACE (Ambion by Applied Biosystems, Darmstadt, Germany) kit was used as described in the manufacturer's protocol. In brief, after the CIP-treatment RNA was purified using an Acid-Phenol-Chloroform extraction. Tabacco Acid Pyrophosphatase (TAP)-treatment, ligation of the 5'-RACE-adapter and the convertion into cDNA by using random hexameres was done as recommended. For the nested PCR reaction *L1CAM *exon1 specific reverse primers were used. L1-exon1 outer rev 5'- ttcctcggggatctggataa-3', L1-exon 1 inner rev 5'- tagtatCTCGAGggaggagaggccacacgta-3'; XhoI binding site in capital letters for cloning into pcDNA3 vector for sequencing purposes. Sequencing reactions were performed at GATC (Konstanz, Germany).

### *In silico *promoter analysis

The sequence used for the *in silico *analysis of transcription start sites and predicted transcription factor binding sites is accessible at [GeneBank:U52112.29]. According to this sequence promoter 1 is located from 42.468nt to 39.083nt (-3385nt) with exon 0 starting at position 39083. Promoter 2 is located at 33.034nt to 28.885nt (-4149nt). HUSAR Bioinformatics lab http://genome.dkfz-heidelberg.de/ software was used to identify TSS. HUSAR accesses different databases to predict TSS like DOOP, CAGE, DBTSS, and MBPromDB. Transcription factor analysis was performed using Alibaba 2.1 software at http://www.gene-regulation.com. In a second step further factors could be analysed using HUSAR database http://genome.dkfz-heidelberg.de. Lef-1 binding sites were identified using Footer 2.0 analysis tool [28-30]. Transcript variant analysis was performed using http://www.ensembl.org.

### Statistical analysis

For the analysis of statistical significance the Student's *t*-test was used. P-values in the figures are indicated as follows: *< 0.05, **< 0.01 ***< 0.001.

## Results

### β-catenin and Slug can regulate L1CAM in endometrial carcinoma

**Figure 1:**
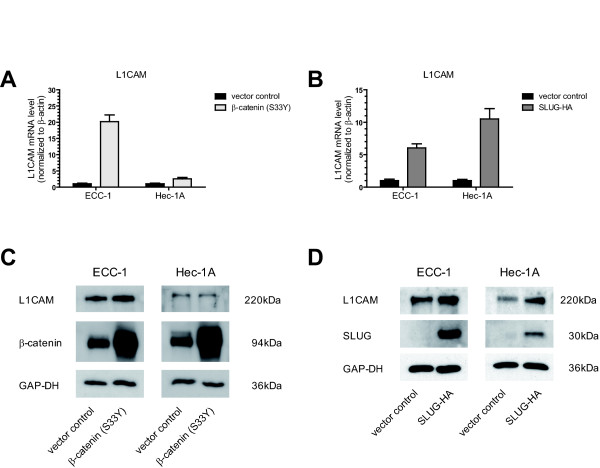
**L1CAM mRNA and protein levels in endometrial carcinoma cell lines overexpresssing transcription factors β-catenin or Slug**. (**A**) Regulation of L1CAM expression after transfection of a point mutated, constitutively active form of β-catenin (S33Y) or control pcDNA3 plasmid as determined by qRT-PCR using specific primers. (**B**) Regulation of L1CAM levels after overexpression of a HA-SLUG or control plasmid. Means ± SD from n = 3 independent experiments are shown. (**C**) Western blot analysis for L1CAM and β-catenin as well as GAP-DH, used as a loading control. (**D**) Western blot analysis for L1CAM and anti SLUG as well as GAP-DH, used as a loading control. A representative experiment from n = 3 is shown.

Recent papers suggested a role of the transcription factors Slug and β-catenin in *L1CAM *regulation [12,24,26]. To confirm a role of these transcription factors in EC, we examined whether overexpression of Slug or a stabilised, constitutively active form of β-catenin (S33Y) in EC cell lines resulted in augmented L1CAM levels. Indeed, β-catenin (S33Y) overexpression strongly upregulated L1CAM mRNA levels in ECC-1 cells but only a minor effect was observed in Hec-1A cells (Fig. 1A). In contrast, overexpression of Slug showed highest induction of L1CAM in Hec-1A cells (Fig. 1B). These results were confirmed at the protein level by Western blot analysis of transfected cells (Figs. 1C and 1D).

### *In silico *analysis of the L1CAM promoter region

**Figure 2:**
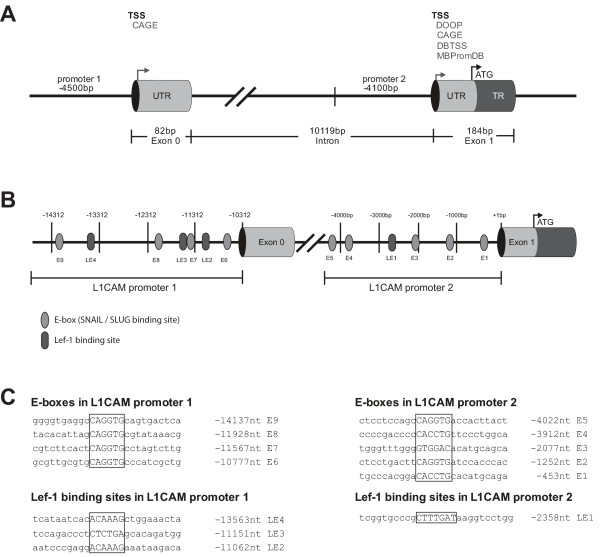
***In silico *characterisation of the L1CAM promoter**. (**A**) Schematic illustration of the L1CAM promoter region according to the Ensembl database. Exon 1 contains the translational start codon (ATG) and a transcription start sites (TSS) predicted by the indicated databeses DOOP, CAGE, DBTSS, MBPromDB. The upstream sequence of appr. 4100 bp was identified as promoter 2. The non-coding Exon 0 (82 bp) is located appr. 10 kb upstream and is followed by an appr. 4.5 kb promoter region (promoter 1). A second TSS is predicted by the program CAGE. (**B**) Schematic illustration of E-boxes and TCF/LEF-1 binding sites in promoter 1 and promoter 2. (**C**) DNA sequences of E-boxes and Lef-1 transcription factor binding sites in promoter 1 and promoter 2.

To further analyse this regulation, we investigated the structure of the *L1CAM *promoter in more detail. Given the conflicting data in the literature, we carried out an *in silico *analysis of putative TSS in the *L1CAM *gene using various bioinformatics tools. As indicated in Fig. 2A, four of the algorithms (DOOP, CAGE, DBTSS, MBPromDB) employed by a HUSAR analysis predicted a TSS in front of exon 1. The CAGE database suggested an additional TSS in front of exon 0. We termed the regions upstream of exon 0 as "promoter 1" and the 5'-sequence in front of exon 1 as "promoter 2".

We next searched for respective binding motifs in the two promoter regions. As indicated in Fig. 2B and 2C we identified several E-boxes for the binding of Slug/Snail as well as binding sites for β-catenin/TCF-LEF (Fig. 2B and 2C). E-boxes were numbered E1-E9 and binding sites for β-catenin/TCF-LEF were labelled as LE1-4 (Fig. 2B). The respective DNA-binding sites are shown in Fig. 2C.

### Identification of in situ transcription start sites by 5'-RLM-RACE

**Figure 3:**
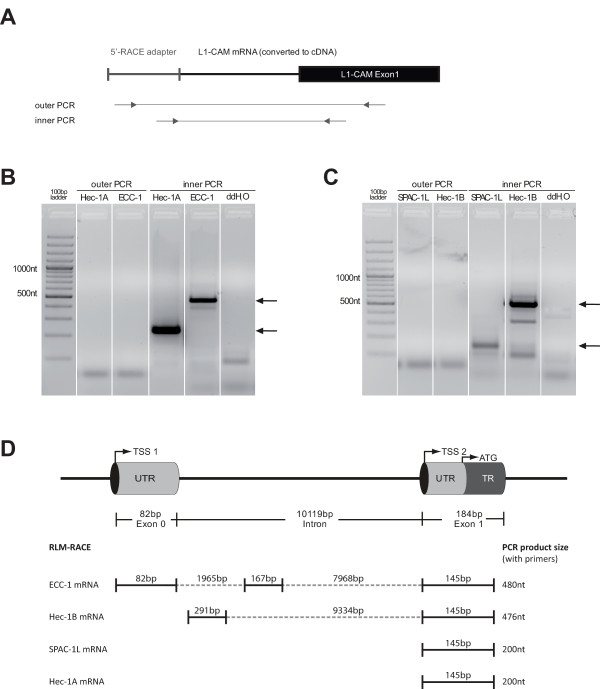
**RLM-RACE to determine transcription start sites in endometrial carcinoma cell lines**. (**A**) Schematic outline of the RLM-RACE reaction consisting of the ligation of the 5'RACE-adaptor followed by 2 nested PCR reactions with outer and inner primers. (**B and C**) RNA preparations of Hec-1A, ECC-1, SPAC-1L, and Hec-1B cells were subjected to the RLM-RACE analysis. On the agarose gel the products of the nested PCR reactions obtained after outer (no product because of low copy number) and inner PCR are shown. Note the different size of amplified products. The water control served as a negative control without template. (**D**) Amplified bands were excised from the gel and subjected to DNA sequencing. The resulting products and the respective TSS are schematically indicated.

Next, we investigated the site of transcriptional initiation of the human L1CAM gene in endometrial carcinoma cell lines. Therefore, we selected the L1CAM positive cell lines ECC-1, Hec-1A, Hec-1B and SPAC-1L and determined the TSS using RLM-RACE technique. In brief, the mRNA from each cell line was decapped and an 5' RACE adaptor was ligated to the 5'-mRNAs. The 5'-ends were amplified in two consecutive PCR reactions (see Fig. 3A) and the products were separated by gel electrophoresis (Fig. 3B and 3C). The resulting fragments varied between 150 and 500 bp, were subcloned and sequenced. In ECC-1 cells the longest transcript comprised exon 0, a 167 bp sequence derived from the intron 1 followed by the coding exon 1 (Fig. 3D). In Hec-1B cells, the exon 0 was clearly not present but instead a 291 bp fragment of intron 1 directly followed by exon 1 was identified. In addition a weaker byproduct of 300 bp was detected that upon sequencing was unrelated to *L1CAM*. In SPAC-1L and Hec-1A cells the 5'-end of the mRNA started with exon 1. Thus, the TSSs for *L1CAM *transcripts are variable in EC cell lines.

### Luciferase activity assays for promoter 1 and 2

**Figure 4:**
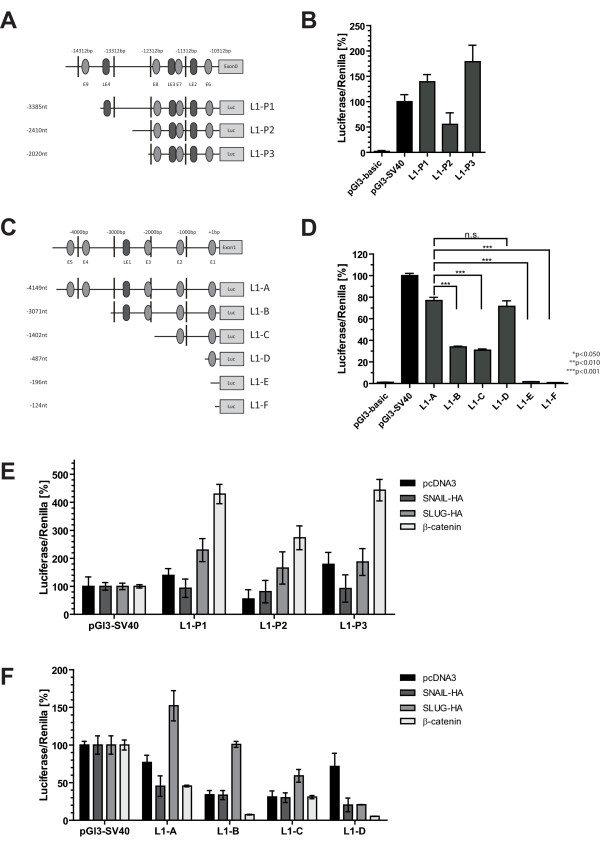
**Luciferase reporter assays for L1CAM promoter 1 and promoter 2**. **(A and B**) Schematic illustration of luciferase promoter constructs of variable length. The E-boxes (E1-E9) and LEF-1 binding sites (LE1-LE4) have been labelled in a consecutive fashion and are indicated. Note that the constructs contain variable number of E-boxes and LEF-1 binding sites and are referred to as L1-P1 to L1-P3 (from promoter 1) and A-F (from promoter 2). **(C and D) **Luciferase activity assay in Ishikawa cells using the indicated promoter constructs. Values have been normalized to the internal control (renilla) to account differences in transfection efficiency. The pGl3 vector was used as negative control and pGl3-SV40 as positive control. Luciferase activity was determined after 48 h. A representative experiment from each n = 4 is shown. **(E) **Luciferase activity assay of promoter 1 reporter constructs L1-P1 to -P3 in Ishikawa cells using the indicated constructs in combination with overexpression of Snail-HA, Slug-HA, stabilised β-catenin or pcDNA3 negative control. (**F**) Luciferase activity assay of promoter 2 reporter constructs A-D in Ishikawa cells using the indicated constructs in combination with overexpression of Snail-HA, Slug-HA, stabilised β-catenin or pcDNA3 negative control. Data ± SD of a representative experiment from n = 3 is shown. (*** p ≤ 0.001, **p ≤ 0.01, *p ≤ 0.05, n.s. not signicicant).

We next asked whether both promoter regions were functionally active. We constructed a series of luciferase-reporter plasmids covering approximately. 3.5 kb upstream exon 0 (constructs L1-P1-P3) (Fig. 4A). Likewise, similar constructs were generated encompassing approximately 4.0 kb upstream of exon 1 (constructs L1-A to L1-F) (Fig. 4C). Activation of transcription was determined after transient transfection into Ishikawa EC cells that do not express L1CAM. Therefore, a competition for transcription factors with endogenous L1CAM expression can be excluded [24]. Luciferase activity with 50-80 -fold higher levels as compared to the promoter-less control plasmid (pGl3-basic) was recorded for L1-A to L1-D whereas the constructs L1-E and -F did not show activity (Fig. 4D). Interestingly, the activity of L1-A was quite similar to L1-D although a sequence of nearly 3.5 kb had been deleted. Thus, a 487 bp sequence upstream of exon 1 was sufficient to initiate transcription (see below). Promoter 1 constructs (L1-P1 to L1-P3) also gave high luciferase signals with strongest activity of L1-P1 and -P3 constructs (Fig. 4B).

Next, we co-transfected promoter 1 reporter constructs together with expression plasmids for Snail, Slug and stabilised β-catenin (S33Y). As shown in Fig. 4E, β-catenin co-expression increased the promoter activity up to 4-fold compared to the empty pcDNA3 vector. The enhanced activity was seen for all constructs from L1-P1 to -P3 (Fig. 4E). There was also an activating effect (2-3 fold) by Slug overexpression on the activity of L1-P1 and -P2, respectively (Fig. 4E). Interestingly, overexpression of Snail rather inhibited activity and this effect was most prominent for the L1-P1 and -P3 constructs.

A similar analysis carried out on promoter 2 constructs L1-A to L1-D showed a different pattern (Fig. 4F). Compared to the empty pcDNA3 vector control, only Slug co-transfection showed increased activity on L1-A, -B and -C constructs. In contrast, overexpression of β-catenin or Snail, respectively, resulted in weak inhibitory or no effects (Fig. 4F). All co-transfected plasmids were inhibitory for the minimal promoter construct L1-D (Fig. 4F).

### Snail/Slug ratios determine activation or inhibition

**Figure 5:**
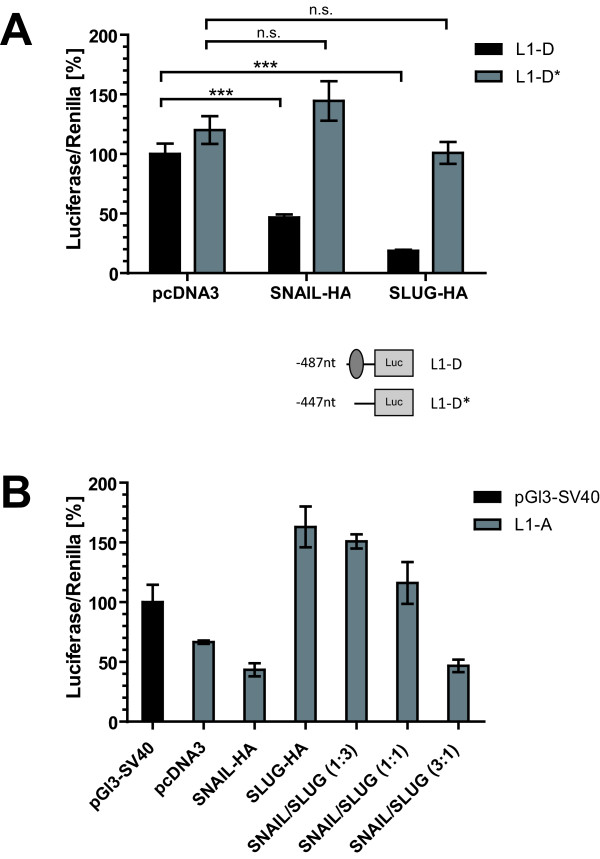
**Transcription factor overexpression and L1CAM promoter luciferase activity**. **(A) **Demonstration that E-boxes are essential for promoter 2 activity. In the minimally active promoter construct pGl3-L1-D the E-box (E1) was deleted and analysed in Ishikawa cells for promoter activity. (**B**) The ratio of Slug/Snail determines the outcome of promoter 2 activity. The pGl3-L1-A promoter constructs was co-transfected with different ratios of Slug in Ishikawa cells and promoter activity was determined after 48 h. Data ± SD of representative experiments from each n = 3 is shown. (*** p ≤ 0.001, **p ≤ 0.01, *p ≤ 0.05, n.s. not signicicant).

Snail and Slug are known to bind E-boxes with different binding affinities [31]. To further study the opposing effects of Snail and Slug we investigated the minimal promoter 2 reporter plasmid L1-D. Co-transfection of Snail or Slug expression plasmids with the reporter construct led to suppression of luciferase activity (Fig. 5A). When the remaining E-box in L1-D was deleted (L1-D*), the suppressive activity vanished (Fig. 5A).

In contrast, the previous results with longer reporter constructs had indicated that both transcription factors had rather opposing effects. Slug overexpression was found to activate whereas Snail was found to suppress promoter activity (see Fig. 4E &4F). To study whether both transcription factors can compete for binding to the E-boxes we over-expressed various ratios of Snail and Slug. The co-transfection of the L1-A reporter plasmid with an excess of Slug resulted in activation of luciferase activity but this effect was reversed when Snail was in excess (Fig. 5B).

### Differential promoter usage after TGF-β1 treatment

**Figure 6:**
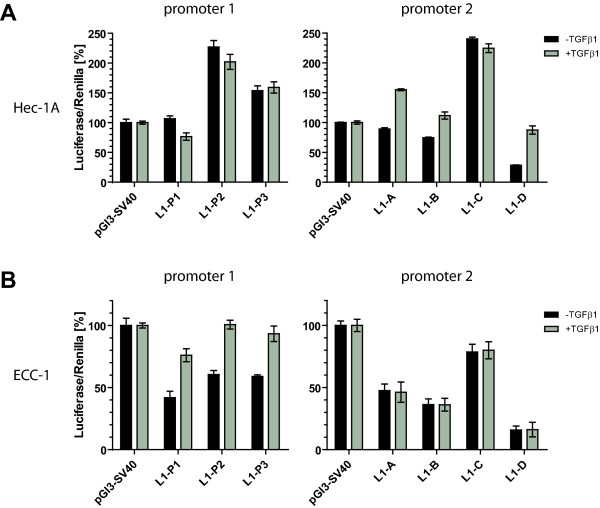
**Differential regulation of TGF-β1 treatment on both L1CAM promoter regions**. Hec-1A and ECC-1 cells were treated for 5 days with TGF-β1 (10 ng/ml) prior to luciferase transfection. Luciferase levels were analysed 48 h after transfection using a dual luciferase assay. (**A**) Hec-1A cells show no increased luciferase activity upon stimulation with TGF-β1 on promoter 1, but on promoter 2. (**B**) ECC-1 cells show an enhanced luficerase activity on promoter 1 when pretreated with TGF-β1, but not on promoter 2. Data ± SD of a representative experiment from n = 2 is shown.

We have shown before that TGF-β1 treatment of ECC-1 and Hec-1A induced an EMT-like phenotype, i.e. downregulation of the epithelial marker E-Cadherin and up-regulation of vimentin but also L1CAM [24]. In order to analyse which *L1CAM *promoter sites might be used in this process, both cell lines were treated for 5 days with TGF-β1 and subsequently transfected with luciferase reporter constructs. In Hec-1A cells, only promoter 2 constructs (L1-A, -B and -D) showed enhanced activity after TGF-β1 treatment (Fig. 6A). Conversely, in ECC-1 cells only promoter 1 constructs (L1-P1, -P2, -P3) revealed augmented activity (Fig. 6B).

### Chromatin-IP analysis for promoter binding

To demonstrate the binding of β-catenin/TCF-LEF complex or Slug to both cellular promoter regions, we carried out a Chromatin-IP analysis. ECC-1 and Hec-1A cells were transfected with pcDNA3 control or stabilised β-catenin plasmids and Chromatin-IP was carried out using an anti-β-catenin specific mAb. Likewise, cells were transfected with control or a Slug-HA plasmid, respectively, and Chromatin-IP was carried out using an anti-HA mAb. To quantitatively analyse the binding of the transcription factors to the predicted binding sites, the precipitated chromatin was analysed using qRT-PCR with primers specific for the selected regions.

**Figure 7:**
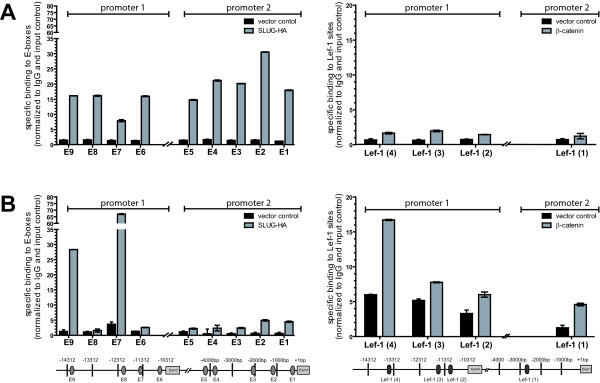
**Chromatin-IP analysis of promoter 1 and promoter 2 sites**. Hec-1A (**A**) and ECC-1 (**B**) cells were transfected with HA-Slug (left panels) or β catenin (right panels) or an empty vector control (pcDNA3). After 48 h cells were fixed, genomic DNA was isolated sheared for Chromatin-IP using antibodies to β catenin or HA. Liberated DNA was subjected to qRT-PCR analysis using primers specific for all E-boxes or Lef-1 binding sites in the L1CAM promoter 1 or promoter 2 regions. Data ± SD of a representative experiment from n = 3 is shown.

In Hec-1A cells the overexpression of Slug-HA led to a strong binding of Slug to the identified E-boxes (Fig. 7A, left panel). Slug binding to promoter 2 was stronger than to promoter 1, with highest binding to E-box 2. The β-catenin overexpression in Hec-1A cells led only to a small increase in binding to the Lef-1 sites. The constitutive level of bound β-catenin in vector control transfected cells was also relatively low (Fig. 7A, right panel). In ECC-1 cells overexpression of Slug only resulted in an enhanced binding to two of the identified E-boxes (Fig. 7B, left panel), where the E-boxes E9 and E7 in promoter 1 were specifically and exclusively bound by Slug. In ECC-1 cells, the Lef-1 sites were constitutively more occupied than in Hec-1A cells, and after overexpression of β-catenin a strongly increased binding to all Lef-1 sites was detectable. Strongest binding was seen at the LE4 site in promoter 1 (Fig. 7B, right panel).

For specificity control we confirmed that all immuno-precipitated DNAs showed no product with off-target control primers (not shown). This suggested that the identified binding sites in the *L1CAM *promoter 1 and promoter 2 regions were specifically occupied by the respective transcription factors.

### L1CAM expression is correlated with high levels of Slug in EC lines

**Figure 8:**
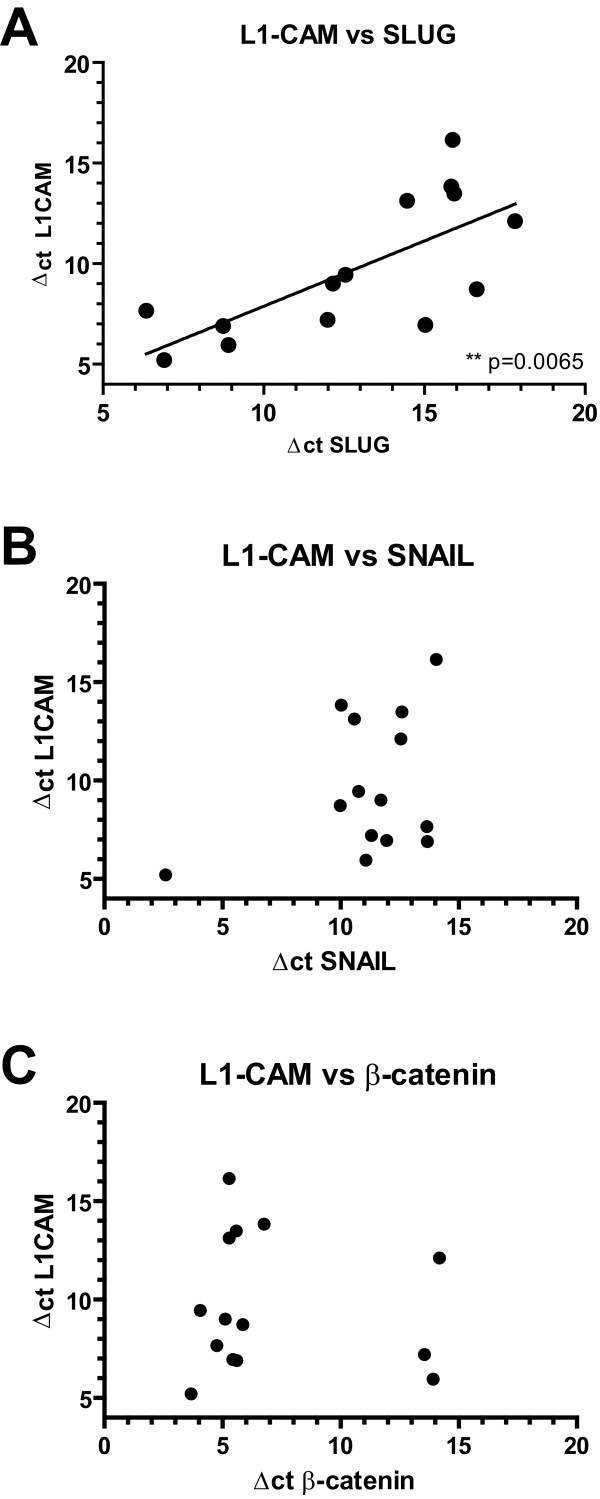
**Correlation of L1CAM and various transcription factors in EC cell lines**. Δct values using β-actin as standard were determined by qRT-PCR analysis in each cell line for L1CAM, Slug, β-catenin and Snail. The EC cell lines included were: Ishikawa, AN3CA, ARK 1, ECC-1, Hec-1A, Hec-1B, Hec-155, IK ER^-^, MFE 319, RL-95 2, SNG II, SNG M, SPAC-1L, SPAC-1 S. (**A**) L1CAM versus Slug. (**B**) L1CAM vs. Snail; (**C**) L1CAM vs. β-catenin. Note that low Δct values mean high expression of the respective mRNA. A representative experiment from n = 3 is shown.

We correlated L1CAM mRNA expression levels with those of β-catenin, Slug and Snail in a panel of 14 EC cell lines [24,27]. For each cell line we determined the Δct value of all four markers. Interestingly, when the levels of Slug expression were plotted vs. L1CAM a significant correlation was found (p = 0.0065) (Fig. 8A). There was no correlation observed between L1CAM and β-catenin or Snail expression (Fig. 8B and 8C). These results support the conclusion that Slug rather than β-catenin is involved in the regulation of L1CAM in cultured EC cell lines.

## Discussion

Recent data have shown that the expression of L1CAM in tumours is upregulated when EMT is induced by TGF-β1 treatment [24,26]. To better understand this process, we have now analysed the human *L1CAM *gene in more detail. We provide evidence that the *L1CAM *gene has two promoter regions that are both active in luciferase reporter assays. Our data suggest that both predicted TSS can initiate transcription utilizing the alternative promoter region in a cell line specific manner. By co-expression studies we could show that promoter 1 is mostly responsive to β-catenin overexpression (and to a minor extent to Slug) whereas promoter 2 responds to Slug only. Despite the ability of β-catenin to activate promoter constructs in activity assays, the expression of L1CAM in EC cell lines seems to dependent mostly on Slug. These results provide new insights in the transcriptional regulation of L1CAM during tumourigenesis.

We initially used a bioinformatics approach to identify promoter regions in the *L1CAM *gene as well as RML-RACE experiments to determine the TSS of *L1CAM *specific transcripts. We identified two putative promoter regions in the human *L1CAM *gene. Earlier studies had already described a promoter region in front of the non-translated exon 0 located > 10 kB upstream the ATG site [21]. For ECC-1 cells we confirmed the presence of a TSS in front of exon 0 similar to the reported TSS in mouse N2A neuroblastoma cells and mouse brain [21]. In addition, in ECC-1 cells we observed an additional exon of 167 bp that had not been identified before. Interestingly, in Hec-1B cells a shorter transcript lacking exon 0 was identified. Whether this band is a product of alternative transcription initiation is presently unknown. In EC cell lines such as Hec-1A and SPAC-1L the TSS was localised directly in front of the ATG site consistent with an additional promoter site adjacent to exon 1. In neural tissues and cell lines the latter promoter site has also been described [20]. When promoter 1 and promoter 2 reporter constructs were assayed in Ishikawa cells, both regions were found to be functionally active. Thus, our results obtained in human tumour cell lines recapitulate the findings in neural cells and demonstrate that the human *L1CAM *gene has two active promoter elements that are differentially used.

We identified several binding sites for β-catenin/TCF-LEF and E-boxes for Slug/Snail in promoter 1 and 2. The putative binding sites are unevenly arranged and β-catenin/TCF-LEF sites are mostly located in promoter 1 whereas E-boxes concentrate in promoter 2. Overexpression of Snail resulted in suppressed reporter activity. This suppression can be counteracted by increasing amounts of Slug as shown by the ratio experiment.

Overexpression of stabilised β-catenin with promoter 1 reporter plasmids resulted in up to 4-fold activation that is consistent with earlier studies [12]. Co-transfection of Slug resulted in a minor activation. In contrast, coexpression of Slug activated efficiently promoter 2 reporter constructs whereas no activating effect of β-catentin was seen. The pattern of differential reactivity was also noted in Chromatin-IP experiments. Here, Slug overexpression was most efficient in Hec-1A cells (promoter 2 dependent) whereas stabilised β-catenin was significantly more active in ECC-1 cells. Finally, overexpression of stabilised β-catenin augmented L1CAM expression only in ECC-1 cells whereas Slug overexpression activated *L1CAM *in both ECC-1 and Hec-1A cells. Collectively, these results demonstrate a differential usage of promoter sites in the L1CAM gene and support a role of β-catenin and Slug in the regulation of L1CAM expression.

Earlier work has suggested a pivotal role of β-catenin for the regulation of L1CAM [12]. This conclusion was based on a couple of consistent observations. First, the authors showed a mouse reporter plasmid (representing promoter 1 sequences) with an enhanced activity when co-transfected with β-catenin. They could inhibit the *L1CAM *transactivation of the *L1CAM *promoter construct by a cytoplasmic tail of E-cadherin, that binds and sequesters β-catenin from binding to TCF-LEF and by a dominant-negative LEF-1. Further, a siRNAs-mediated knock-down of β-catenin in colon cancer cell lines suppressed β-catenin levels and concomitantly decreased L1CAM expression. Finally, they showed that β-catenin-TCF/LEF-1 complexes could bind in electrophoretic mobility shift assays to an oligonucleotide derived from the *L1CAM *promoter region [12]. These observations were interpreted as a direct effect of β-catenin/TCF on the *L1CAM *promoter [12]. However, it is quite known that *Slug *is a direct target of β-catenin signalling [29], [30] and therefore regulatory effects seen by β-catenin could also be indirect. It is feasible that β-catenin first up-regulates Slug that then in turn activates *L1CAM *expression. A prominent role of Slug in L1CAM regulation was previously suggested in two other studies [26,24] and is supported by our analysis of a panel of EC cell lines that differ in L1CAM expression. However, it should be kept in mind that the earlier data were obtained in colon cancer and that fundamental differences may exist between colon cancer and endometrial cancer. Moreover, mRNA levels of β-catenin might not fully reflect catenin activity in the cells. Our findings suggest that Slug plays a major role in the regulation of *L1CAM *in endometrial cancer and pancreatic cancer [26,24]. It is possible, that under EMT conditions augmented levels of Slug suppress cell-cell adhesion by down-regulating E-cadherin [31] and up-regulation of L1CAM expression resulting in enhanced cell motility and invasion [24,26].

An important question is which factors regulate the promoter usage of the *L1CAM *gene? A recent study on L1CAM expression in colorectal cancer has shown that the *L1CAM *promoter regions (including promoter 1 and 2) are subject of comprehensive changes in DNA methylation of CpG islands [32]. This was seen between different cell lines but also between tumour and normal tissue of 5 different patients [32]. Moreover, treatment of cells with the demethylating agent Azacytidine could alter L1CAM expression in 1 out of 4 cell lines tested. There is also evidence that histone deacetylase inhibitors can up-regulate L1CAM expression [33]. Thus, it is likely that epigenetic factors may have a strong impact on the regulation of L1CAM and possibly on the usage of the two promoter regions.

## Conclusions

In summary, our results show for the first time that L1CAM expression in tumours is regulated by two distinct promoter regions in the *L1CAM *gene and that Slug is a relevant transcription factor for its regulation. The up-regulation of L1CAM during the EMT process underscores the important role that this molecule plays in tumour progression.

## Abbreviations

**EC**: endometrial carcinoma; **EMT**: epithelial-mesenchymal transition; **L1CAM**: L1 cell adhesion molecule; **TSS**: transcription start site; **CIP**: calf intestine phosphatase; **RLM-RACE**: RNA-Ligase-Mediated Rapid Amplification of cDNA Ends

## Authors' contributions

MP cloned the reporter plasmids, designed and performed experiments shown in Figs. 1, 2, 3, 4, 5 and 6 and participated in writing the manuscript. US and MP designed experiments shown in Figs. 7 and 8, US carried out the analysis. CG, SSM and HS participated in the experimental design and in writing and critically reviewing the manuscript. PA supervised the study and the experimental design and wrote the manuscript. All authors read and approved the final manuscript.
